# Evaluation of Health Risk Level of Hand-Arm and Whole-Body Vibrations on the Technical Operators and Equipment in a Tobacco-Producing Company in Nigeria

**DOI:** 10.1155/2019/5723830

**Published:** 2019-12-23

**Authors:** Oluseyi Adewale Orelaja, Xingsong Wang, Dauda Sh. Ibrahim, Umer Sharif

**Affiliations:** ^1^School of Mechanical Engineering, Southeast University, Nanjing, China; ^2^Department Mechanical Engineering, Moshood Abiola Polytechnic, Abeokuta, Ogun State, Nigeria

## Abstract

Vibration is experienced when a body is subjected to either internal or external forces which cause oscillation, with most operators of industrial equipment often exposed to high dosage, higher than the stipulated values. In this research, Digital Real-Time Frequency Analyzer (RSA 5106A) was used, while the results obtained were evaluated and compared with the health guidelines of the ISO 2631-1 : 1997 and ISO 2631-5 : 2004 standards, as described in the Health Guidance Caution Zone for a daily exposure action value (EAV) of 0.47 m/s^2^ and a daily exposure limit value (ELV) of 0.93 m/s. High acceleration was mostly seen on the *z*-axis in all the results obtained, whereas many were not within the HGCZ (*A*_rms_ <0.47, and >0.93 m/s^2^). Comparing (VDV <8.5 m/s^1.75^ and >17 m/s^1.75^) with the ISO standard, the accelerations on all *x-* and *y*-axes were slightly within the HGCZ, with just a little below 0.47 m/s^2^ limit. The results obtained clearly showed that urgent action is needed virtually on all the equipment in both the Secondary Manufacturing Department (SMD) and Primary Manufacturing Department (PMD) to minimize vibration exposure on the technical operators.

## 1. Introduction

Due to increase in industrial developments, most operators are exposed to vibration at work place every day. Vibration in humans can either be whole-body vibrations (WBVs) or hand-arm vibrations (HAVs). WBV resulted from the operation of different equipment in the manufacturing industries such as food production, mining and construction, agriculture, and general running equipment either while sitting, resting on the back seat in vehicles, or standing on them [1], and it causes discomfort, in-efficiency due to fatigue, and serious health hazards. HAVs occur due to mechanical vibration or shocks because of impact forces applied on the hand-arm surfaces through the whole palm or fingers, for example, vibrations from grinding machines, power tools, ramming machines, or any impacting tools [2]. It is believed that whole-body vibration is a health risk hazard which could cause low back and neck pain. High exposure to WBV and undesirable posture are seen as main causes of musculoskeletal pains among the technical operators and drivers [3]; as a result of greater demand in production volume, many operators work throughout the year with little or no break or interruption in all working hours in a day, throughout the week, and with few days break in 365 days in a year; occupational vibration was subjected to accumulation of vibration energy transmitted throughout the daily working hours [4]. Generally, most operators of machines and vehicles face serious musculoskeletal disorders (MDSs), due to overexposure to vibration [5]. Statistical data from the United States in the year 2015 reveal that the tremendous incidence rate of MSDs in the mining sector was about 12.9% of 10000 mining workers fully engaged in their work, which is more than the average rate when compared with all industries [6]. There are several factors that cause a high vibration effect on operators; they are types of mechanical machine design, maintenance culture, seat type, foundation type, and equipment speed, and we can also consider the type of task being performed on the machine, individual characteristics (operators anthropometry and their posture), condition at work, and production work layout can possibly affect operators overexposure to vibration. Technical operators handling industrial equipment in most industries are mostly subjected to a high intense level of both hand-arm vibrations (HAVs) and whole-body vibrations (WBVs) according to Zhao et al. [7]; as a result of prolonged use of vibrating equipment, this however reduces the efficiency of the machine operators and pose undesirable effects on their well-being and safety, and if not controlled will consequently cause physiological effects such as stroke, osteoporosis (low bone density) due to calcium loss in the hand bone, joint pain, and white patches on the skin, as well as affecting the blood circulatory rate in the human system [8]. According to Eurofound [9], it was also revealed that majority of industrial workers across the world are exposed to different types of vibrations either from tools or machinery, with further analysis revealing that one in every four is exposed at least to a quarter of their working time, and this remained unchanged over the last three surveys conducted in years 1996, 2000, and 2005. Further research has shown that WBV at 2–20 Hz frequencies and at an average intensity of 0.1–2 m/s^2^ could result in general cardiopulmonary effect which increases respiratory and heart rates, cardiac output, and pulmonary ventilation and subsequently minimize oxygen intake and possibly cause operators to slump or feel dizzy [1] during operation. Vibration frequency of 3–6 Hz in the thoracic region can cause nausea, while vibration resonance of about 20–30 Hz frequencies on the head and neck could cause posture instability. Summarily, all these contribute to physiological effects on operators in the tobacco sector, which this research is based on, and thus retards concentration, causing high discomfort and fatigue on all the operators [10, 11].

The work process in most factories such as tobacco industries is always constant (they are mostly not routinized) and highly repetitive, with one technical operator doing the same work for a couple of years without any health check and nonfree and nondiscretionary work breaks [11, 12]; they are exposed to a mixture of work activities, including exposure to hand-arm and whole-body vibrations coupled with a high mentally tasking job by Jahncke et al. [13]. However, this poor design conventional production process structured encourage constant work cycles, causing the operator to spend a longer time on the machine because of no substitute or assistance from anyone and because the company is trying to save so much cost on human management, thereby allowing an operator to do a work that is supposed to be done by two or more operators, not considering the effect of this overexposure and fatigue over a period of time. The objective of this research is to assess and evaluate the level of havoc because of high vibration exposure on the operator and to recommend a long-lasting solution to these effects. There are certain parameters that should to be considered when dealing with vibration measurement, and they include mechanical, biological, and psychological parameters, according to the International Standard for Organization [14]. The ergonomics principle in the design of work systems to minimize vibration and repeated shocks should be in the frequency range of 0.5 Hz to 80 Hz, and this should be strictly adhered to by all industries according to the Directive 2002/44/EC of the European Parliament and of the Council as the Minimum Health and Safety requirements regarding the exposure of workers to risks due to physical agents (vibration), minimum acceptable requirements for the protection of workers in relation health, and safety at work, as stipulated by NIOSH, 1997. Both WBV and HAV can be measured and analyzed as suggested by the ISO standard [14] in two different ways: it can used for prediction of negative health effects on the operators from the maximum value of frequency-weighted vibration exposure based on the directional motion on each of the axis on the surface of the seat or back rest when the crest factor is less than <9.0, the weighted root mean square (RMS) (in m/s^2^) is definitely adequate for evaluation of vibration exposure; secondly, it can also be used on the vibration dose value (VDV) (in m/s^1.75^) when the subjected shock effect on a mechanical member is having a crest factor greater than >9.0, as stated in [12]. Furthermore, the ISO standard [15] gives more measurement guidelines for analyzing vibration in the case of varying numbers of multiple mechanical shocks or sudden impact force on the mechanical member, and this involves the mathematical analysis of cumulative acceleration of dose (*D*_*k*_) and daily static compression dose value (*S*_ed_(8) measured in MPa). If  *S*_ed_ is less than 0.5 MPa, then there will be a minimal tendency of having serious health problems throughout the whole length of time (years) spent at work while being exposed to vibration or shocks [16, 17]. For more understanding, the word “action limit is to ascertain the minimal limit exposure levels which is applicable to the two standards mentioned above. Vibration dose value (VDV) of the frequency-weighted accelerations, as proven in [18], will be measured on all operators and equipment in the Primary Manufacturing Department (PMD) and the Secondary manufacturing Department (SMD), to assess the potentially harmful levels of whole-body vibration and hand-arm vibration. Furthermore, the total exposure period of any operator in a tobacco company needs to be referenced to the Health Guidance Caution Zone, according to International Standard Organization [14] and further reviewed on the long-term consequences of vibration, and the health effect caused by exposure to vibration is in proportion to extent of its intensity [19, 20].

## 2. Materials and Methods

### 2.1. Experimental Instrumentation

**Figure 1:**
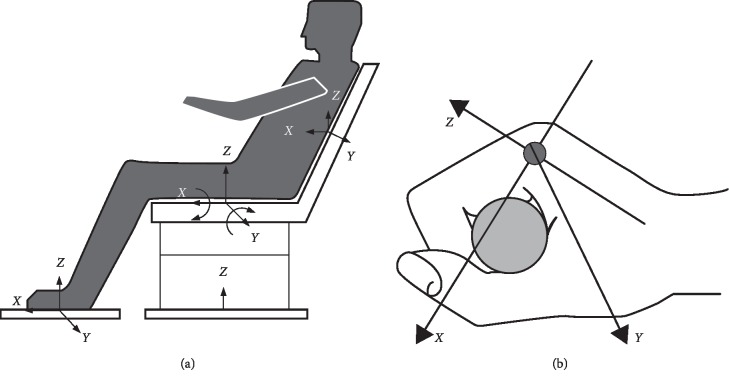
(a, b) The vibration transmissibility coordinate measurement of operator exposure to whole-body and hand-arm vibrations in accordance to [14].

A cross-sectional survey of randomly selected operators at the production floor in two different departments, i.e., in the Primary Manufacturing Department (PMD) and the Secondary Manufacturing Department (SMD) of a Tobacco Company in Ibadan City, Oyo State, Nigeria, was carried out by using a vibration meter to measure the resulting vibration from the machines to the technical operators while in operation, and the measurements of the time history and frequency spectrum were carried out with a Digital Real-Time Frequency Analyzer in the frequency range of 0.3–1600 Hz. The transducers were fastened to the standard mount placed between the source of vibration on the running equipment and then on the operators. The WBV measurements were done, while the operators were loading filters on the running equipment on GD121, Cigarette Packer, and on all the equipment under consideration, and this is in accordance to the measuring procedure outlined, which is also applicable to acceleration levels measured on the operators, and also HAV was measured while griping some major parts of the machine during the production process to allow the measurements in *x-*, *y-*, and *z-*axes, as shown in Figure 1. The vibration on the operators with reference to machines evaluated in the Secondary Manufacturing Department (SMD) include GD121 Cigarette Maker, X3 and X4 Packer, T-10, and Filter loader, while the vibrations of machines such as tobacco cutters, tobacco tipper, and tobacco steamer with respect to the operators were evaluated in the Primary Manufacturing Department (PMD); however, the vibration exposure according to the EU Directive [20] was issued for clear direction of measured vibration levels, with vibration above the exposure value being referred to as the daily exposure action value set for a daily (8 hour) exposure at a frequency-weighted (RMS) acceleration of 0.5 m/s^2^ (when considering the dominant axis of exposure, 1.4*a*_w*x*_, 1.4*a*_w*y*_,  and 1.0*a*_w*z*_), as indicated in the expression below that the employer should take action to reduce exposure on their technical operators. The vibration magnitudes (m/s^2^) corresponding to the hand-transmitted vibration exposure action and exposure limit value in the 2002 Physical Agents (Vibration) Directive of the European Union [21] is shown in Table 1.

We extend our measurements to 24 hours for both EAV and ELV in HAV, and the standard is mostly measured within the range of 8 hours, which can be seen as 2.50 m/s^2^ for EAV and 5.00 m/s^2^ for ELV, as shown in Table 1.

### 2.2. Theoretical Interpretation of Weighted Vibration Exposure (WBV and HAV)

The total vibrations experienced vertically and horizontally and in the transverse direction are summarily interpreted by the expression in equation (1) to calculate the total vibration value (*a*_v_) for the frequency-weighted vibration (*a*_w_) of whole-body vibration and hand-arm vibration on the operators:

(1) $$\begin{array}{c} a_{\text{v}}={\left(K_{x}^{2}\;a_{\text{w}x}^{2}+k_{y}^{2}a_{\text{w}y}^{2}+k_{z}^{2}a_{\text{w}z}^{2}\right)}^{1/2}, \end{array}$$

where *a*_v_ represents the total value of vibration weighted. *a*_w*x*_, *a*_w*y*_,  and *a*_w*z*_ indicate the weighted vibration levels in three directions.

The assessment of WBV and HAV is based on the calculation of daily exposure A_(8__)_ expressed as continuous equivalent acceleration over an eight-hour period, calculated as the highest (RMS) value, or the highest vibration dose value (VDV) of the frequency-weighted accelerations determined on the three orthogonal axes (1.4*a*_w*x*_, 1.4*a*_w*y*_,  and 1.0*a*_w*z*_), where *K* = 1.4 for both the *X-* and *Y*-axes and *K* = 1.0 for the *Z*-axis, is according to the ISO standard [15].

### 2.3. Vibration Transmissibility

**Figure 2:**
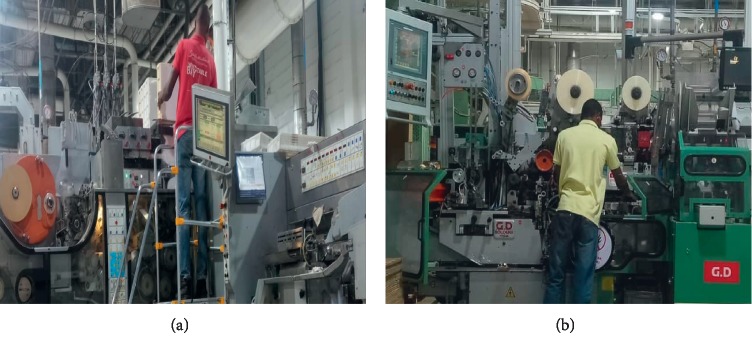
(a, b) Tobacco operator exposure to both WBV and HAV [22].

**Figure 3:**
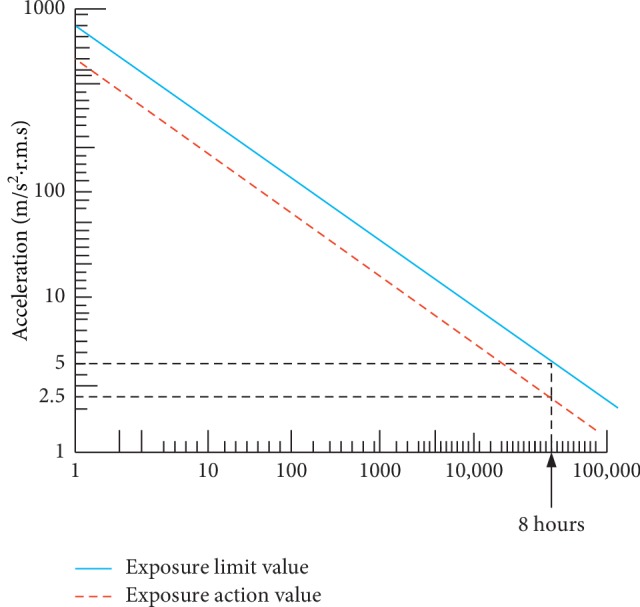
The two “Health Guidance Caution Zones” showing the maximum permissible exposure limit by WBV according to [23].

This is the ratio of the measured WBV or HAV vibration value obtained from the operator, to input vibration on the running equipment. It can be mathematically stated as

(2) $$\begin{array}{c} V_{t}=\frac{\;a_{\left(\text{m},\text{w}\right)\text{v}}}{\;a_{\text{h},\text{v}}}, \end{array}$$

where  *a*_(m, w)v_ = accelerated vibration measured value of WBV or HAV in m/s^2^ under consideration and *a*_h,v_ = measured accelerated value of vibration along the whole body or at hand and arm in *x*, *y*, and *z* directions measured in m/s^2^, having a frequency range of 6.3 to 1250 Hz for HAV and 1 to 80 Hz recommended for WBV. The illustration in Figure 1(a) shows the vibration transmissibility coordinate measurement of operator exposure to whole-body vibration, while Figure 1(b) illustrates hand-arm vibration transmissibility coordinate in accordance to [14]. In addition, Figure 2 indicates the pictorial view of the operator in the production floor exposed to both WBV and HAV, while Figure 3 also shows the maximum permissible exposure limit by hand-arm vibration according to ISO : 5349-2 [23].

### 2.4. Maximum Permissible Exposure Limit

Evaluation of the effects of vibration on health, according to [14], is determined using the frequency-weighted RMS. The overall assessment is usually carried out according to the worst axis of frequency-weighted RMS acceleration (including multiplying factors). Two “Health Guidance Caution Zones” are included in [14] to assist with interpretation of the worst axis of the frequency-weighted (RMS) acceleration in Figure 3. Two zones are provided as they are derived from RMS and VDV approaches. The standard states that “for exposures below the zone, health effects have not been clearly documented and/or objectively observed in the zone, caution with respect to potential health risks is indicated and above the zone health risks are likely.” The zones coincide for durations of about 4 to 8 h, and the standard warns against using the zones for shorter durations. Indeed, for exposure durations between about 5 and 30 min, it is possible to exceed the limits of the zone. For assessments according to VDV, the Health Guidance Caution Zone has upper and lower bounds at 8.5 m/s^1.75^ and 17 m/s^1.75^, respectively.

### 2.5. WBV and HAV RMS Average Acceleration Evaluation

The vibration level experienced by the operators was measured using the calibrated Digital Real-Time Frequency Analyzer (RSA 5106A) with the frequency range of 0.3–1600 Hz, which is within (A8) the stated vibration exposure directives. Acceleration magnitude of WBV and HAV was measured in accordance to [14] standard and European Parliament [21], as seen in equation (3), where the *A*_RMS_ values can be determined by this expression; however, Table 2 also shows the equipment type, number under consideration, speed, and the type of adopted maintenance culture:

(3) $$\begin{array}{c} A_{\text{RMS}}\;=\frac{1}{T}\int_{0}^{T}a_{w}^{2}\left(t\right)\text{d}t, \end{array}$$

where *A*_RMS_ = RMS average acceleration(m/s^2^), *a*_*w*(*t*)_ = acceleration at time *t*(m/s^2^), and T=time of vibration exposure in seconds.

According to Table 3, it shows the exposure values (EVs) and Health Guidance Caution Zone (HGCZ) for the upper and lower limits for both the exposure action value (EAV) and exposure limit value (ELV), weighted root mean square (WRMS), and vibration dosage value (VDV) for the WBV of the tobacco equipment's operators in accordance to ISO 2631-1 (1997) and EU Directive (2002) and static daily compression dose value (Sed (8) measured in MPa); if *S*_ed_ is less than 0.5 MPa, then there will be a minimal tendency of having serious health problems throughout the whole length of time (years) spent at work while exposing to vibration, while *R* is for accessing the effect of the vibration rate in accordance to ISO 2631-5.

### 2.6. Influence of Equipment Maintenance on Operators Vibration Exposure

It is believed that plant aging is due to wear and tear encountered during the production process, and this influences the extent of vibration exposure (WBV and HAV) of the technical operators working on the machines. Therefore, Table 2 shows the maintenance history of each of the equipment which can be used to predict the possible extent of vibration that could be exposed to, by the technical operator.

### 2.7. Vibration Dose Value (VDV)

The technical operators' vibration exposure due to high speed of transfer drums and abrupt cutting of the cigarette into two at the same time coupled with very high speed of electrical machines and pneumatic system was also measured along *x-*, *y-,* and *z*-axes; when the crest factor is greater than 9, the RMS acceleration value will not be enough to evaluate the WBV exposure. The mathematical illustration is shown using the relationship in the following equation:

(4) $$\begin{array}{c} V_{\text{DV}}=\int_{0}^{T}a_{\text{w}}^{4}\left(t\right)\text{d}t. \end{array}$$

In general, the number of exposure points, *P*_E_, is defined as

(5) $$\begin{array}{c} P_{\text{E}}={\left(\frac{Ka_{\text{w}}}{0.5\;\text{m}/{\text{s}}^{2}}\right)}^{2}\frac{T}{8\text{ hours}}*100. \end{array}$$

The daily exposure can be calculated from the exposure point using the following expression:

(6) $$\begin{array}{c} D_{\text{E}}=0.5\;\text{m}/{\text{s}}^{2}\sqrt{\frac{P_{\text{E}}}{100}}\text{ or }a_{\text{w}}\sqrt{\frac{T}{8}}, \end{array}$$

where *a*_w_ is the vibration magnitude in  m/s^2^, *K* is the multiplying factor of 1.4 along the *x*- and *y*-axes and 1.0 along the *z*-axis, and *T* is the exposure time in hours.

### 2.8. Health Effect Assessment

In most industries, vibration exposure assessment procedures were not in accordance to both ISO 2631-1 and EEC Directive 2002/44/EC, especially in the tobacco industry. The assessments should be based on the daily eight hour RMS (A8) level, the vibration dose value (VDV), and vibration exposure points [9] of all the operators and equipment.

**Figure 4:**
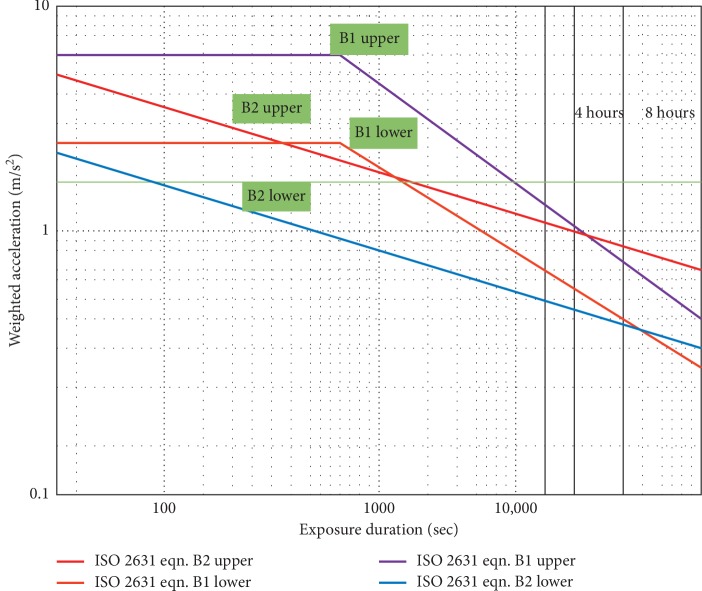
Health Guidance Caution Zones as provided in [14].

To evaluate the health risk levels of vibration according to [14] stipulated that “Health Guidance Caution Zone (HGCZ),” as shown in Figure 4, classified vibration exposures as “unknown,” “possible,” or “likely”. This standard stated two assessment methods (B1 and B2), where B1 depends on a square root of the time relationship spent on the equipment, mostly eight hours (A8), while B2 is based on a fourth root of the time relationship (VDV).  Table 4 further indicated the weighted RMS levels against both the B1 and B2 assessment limits according to [21], and all these needed to be adopted by tobacco industries.

This indicates the analysis test of the weighted RMS levels against both the B1 and B2 assessment limits and reports the health assessment risks, as shown in Table 5.

It must be noted that, in 4–8 hours of exposure, the caution zone is essentially the same for both B1 and B2 when these assessment methods were adopted.

## 3. Results

**Figure 5:**
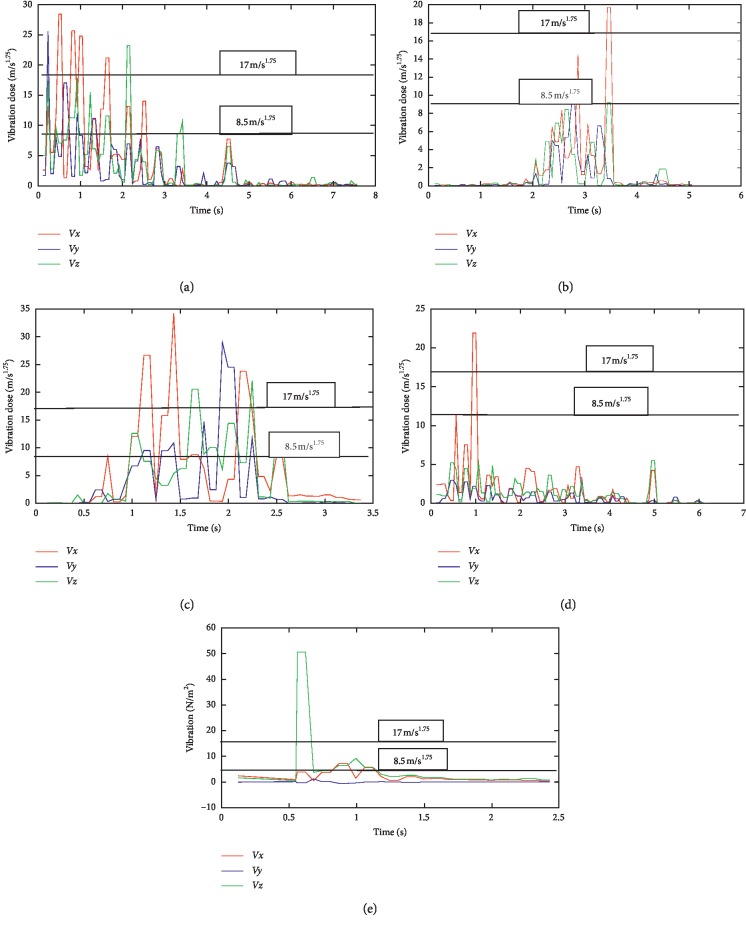
Average vibration dose results on equipment in SMD: (a) vibration on GD121; (b) vibration on X3-X4; (c) vibration on Tray filler; (d) vibration on T10; (e) vibration on Filter loader.

**Figure 6:**
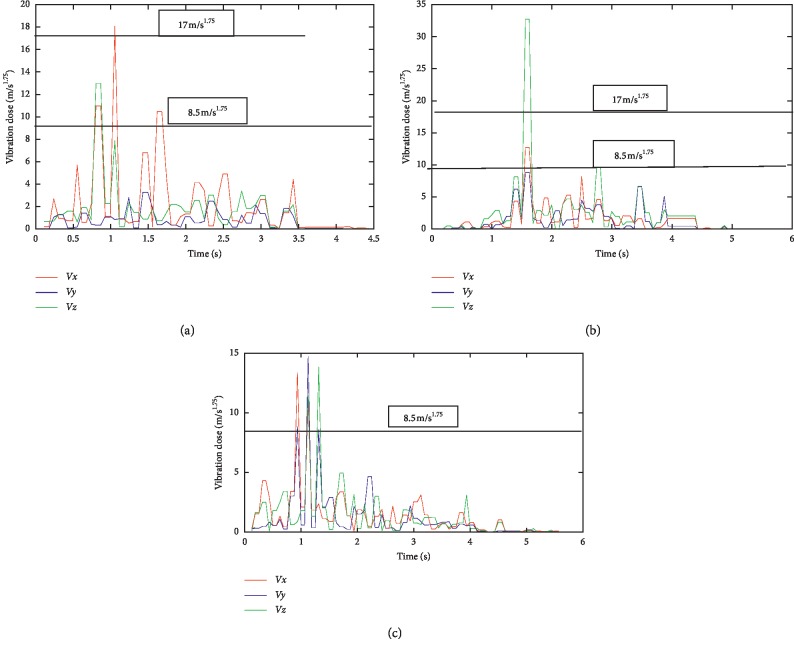
Average vibration dose results on equipment in PMD: (a) vibration on tobacco tipper; (b) vibration on tobacco cutter; (c) vibration on tobacco boiler.

Maintenance details and schedules of all the machines in SMD and PMD were obtained from the maintenance department, with vibration data taken from all the equipment, and processed using excel spread sheet; then, the data were imported using Matlab 2016b and then used to generate the vibration curves on all the axes (*x*, *y*, and *z*). Although records obtained from the maintenance department show that there is bimonthly routine maintenance on all the machines with exceptions of emergency maintenance, which are unavoidable, vibrations on all the equipment are still very higher than the acceptable limit. It was discovered that most of the equipment were old due to enormous vibrations that were detected on them. The comparative vibration dose results obtained from accelerometers placed on all the equipment on SMD are shown in Figure 5, and it shows the vibration dose level on the operator running the following machines: GD121, Tray filler, X3-X4 packer, Filter loader, and T-10 respectively, with the maximum and minimum dose limits of 17 m/s^1.75^ and 8.5 m/s^1.75^ according to the ISO standard, while Figure 6 also shows the maximum and minimum dose limits of 17 m/s^1.75^ and 8.5 m/s^1.75^ from the results obtained from the operators operating tobacco tipper, tobacco cutter, and tobacco boiler in PMD, all in accordance to [22]. It was observed all the equipment in SMD has a higher [22] dose value, as it is all far above the acceptable limit range of 17 m/s^1.75^ (max. limit) and 8.5 m/s^1.75^ (min. limit) although equipment in PMD also has a high [22] value, it is lesser to SMD equipment, which implies that operators in SMD are more prone to vibration risk.

### 3.1. WBV and HAV Evaluation

**Figure 7:**
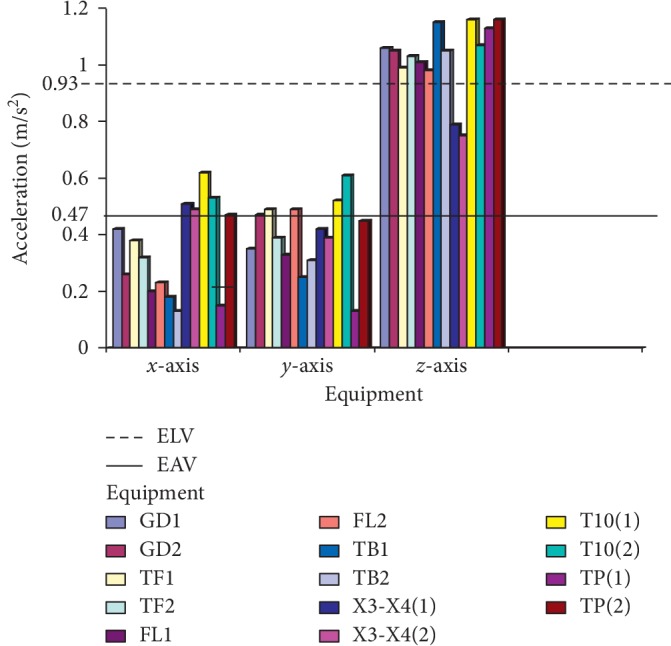
Average measured weighted RMS value of WBV on technical operators in SMD and PMD production floor.

**Figure 8:**
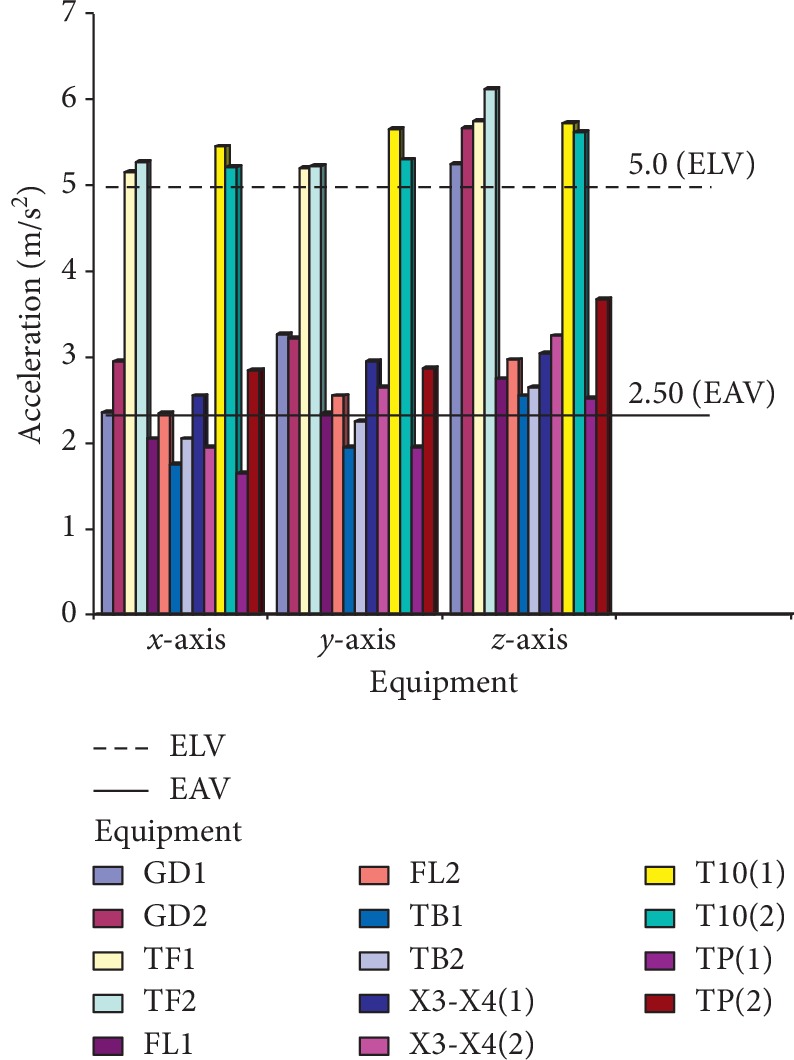
Average measured weighted RMS value of HAV on technical operators in SMD and PMD production floor.

The criteria that are used for the assessment and evaluation of this work are in consistence with the ISO standard [14], and the values of *A*_rms_ on the technical operators were measured, as shown in Figures 7 and 8, where GD1 and GD2 represent the cigarette maker, TF1 and TF2 represent the Tray filler, FL1 and FL2 represent the fork lift, TB1 and TB2 represent the tobacco boiler, X3-X4 (1 and 2) is the Cigarette Packer model, T10 (1 and 2) represents the cigarette box compiler, and TP1 and TP2 represent the tobacco tipper, by comparative analysis as suggested by the ISO standard [14]; the exposure action value (EAV) [24] and exposure limit value (ELV) of 0.47 and 0.93 m/s^2^ must not be exceeded for WBV, as indicated in Figure 7, while EAV of 2.5 m/s^2^ and ELV of 5.0 m/s^2^ must not be exceeded for HAV, as shown in Figure 8; however, it was seen that most of the values for both WBV and HAV obtained in both figures exceeded [24] stated standard limits.

### 3.2. Average Vibration Dose Value (VDV) Computation

The VDV is calculated based on the vibration exposed to along all the three axes (*x*, *y,* and *z*), while the equipment is running within a speculated time. The VDV exposure limit is mostly more than the exposure action limit (EAL) and exposure limit value (ELV) with standard values of 8.5 and 17 m/s^1.75^, respectively, which are the accepted stipulated standard. Figures 7 and 8 show the calculated vibration dose on the equipment.

## 4. Discussion

**Figure 9:**
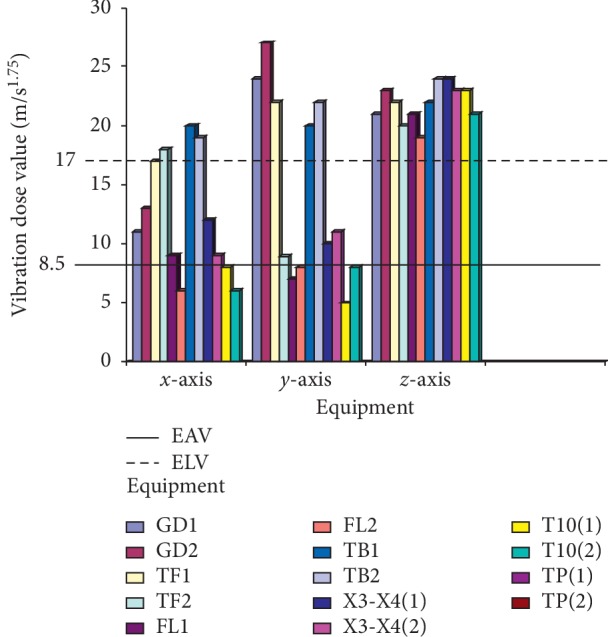
Average vibration dose value of WBV transmitted to the operators by the equipment on SMD and PMD production floor.

**Figure 10:**
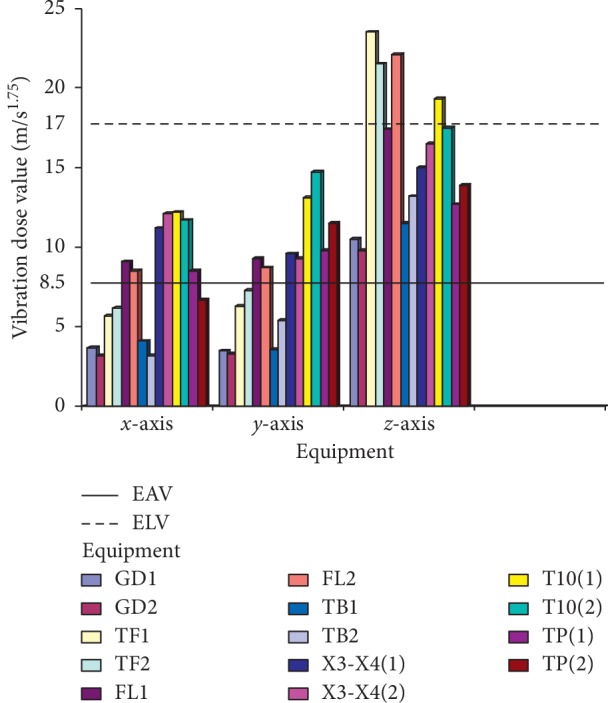
Average vibration dose value of HAV transmitted to the operators by the equipment on SMD and PMD production floor.

**Figure 11:**
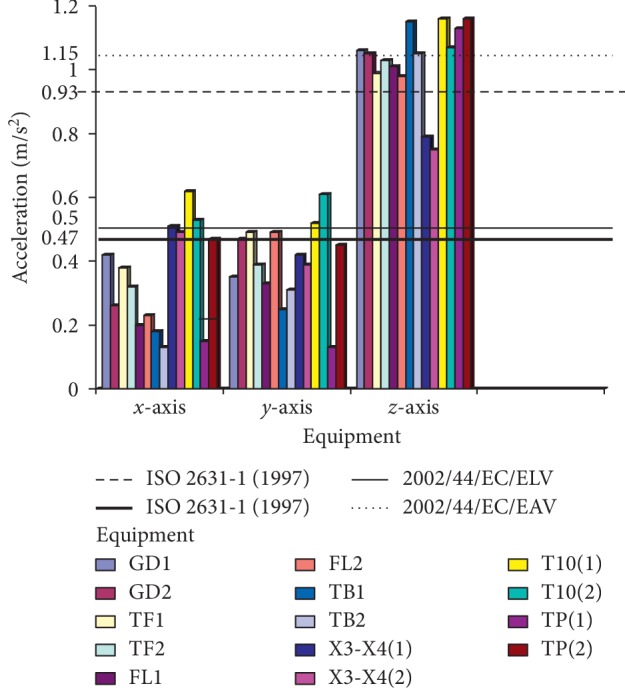
ISO 2631-1 (1997) and 2002/44/EC standard comparison.

No studies about vibration effects on the tobacco operators have been previously reported. In this study, the effect of whole-body and hand-arm vibrations on the operators and equipment was evaluated and analyzed on different parts of the cigarette-producing machine with respect to the combined effect on the discomfort rate caused to the operators. The daily vibration exposure action value according to the European Directive [21] standard value is 0.5 m/sec^2^ [24], which indicated a negative health effect is possible on the operators, and several actions must be taken to minimize the exposure and to alleviate the health risks. From the result, the daily vibration exposure limit value is mostly greater than 1.15 m/sec^2^ for WBV in almost all the equipment which showed that there is a serious vibration threat on the operator; therefore, an urgent action is required to be taken to control the vibration exposure to a level below the ELV, as also suggested by [21]. The data obtained from Figures 5 and 6 indicated that equipment on SMD are tremendously subjected to high vibration since most of the axes (*x*, *y*, and *z*) have values more than the stipulated values of 17 m/s^1.75^. Looking at Figures 7 and 8, the range of values got on the *z*-axis are substantially more than *x-* and *y-*axes, which implies that all the vibrations along the *z*-axis are within the HGCZ; that is, it fell between EAV and ELV, which summarily indicated that all the equipment was generating an extremely high vibration intensity and urgent action are mostly required. In this analysis, mostly *y-* and *z*-axes weighted acceleration are higher than EAV and ELV; therefore, quick action is required to arrest the high exposure needed. While the operators were standing on the floor and holding the most sensitive operational parts while accessing the vibration, HAV is mostly experienced, and the vibration effect on the operator in this case is also high but still less than vibration when the whole body is subjected to WBV vibration. Data obtained in Figures 8 and 9 indicated the evaluated result of VDV, and it is seen that some results on *x-* and *y*-axes are above ELV, while some are within the HGCZ stipulated recommendations, which imply that urgent actions are needed on all the equipment to minimize the amount of vibration on them. It could be seen that most vibration results obtained in this research are more than this stipulated weighted root mean square (RMS) value range of 0.47–0.93 m/s^2^ for WBV, which implies that the vibrations on most of the equipment on the operators are on the extreme, with a clear indication of high health risk. By comparing ISO 2631-1(1997) and 2002/44/EC standard, the variance between the two standards with the 8 hour production work span was discovered that almost all the entire axis has a very high acceleration, which also confirms the operator exposure to severe vibrations. Conclusively, the highest acceleration was mostly seen vertically (*z*-axis), as shown in Figures 10 and 11, which affirmed that the high acceleration values from *x-* and *y*-axes are due to extreme speed of operation of the equipment; since there is a daily production target required to be met, it is a rule that operators must operate at an optimum speed of about 15,000 rpm, which consequently causes high vibration, this can be related to the findings of Wolfgang and Burgess-Limerick [4] and it is also in agreement with the findings of Eger [18], and these high vibration risks effects can also be traced to the inadequate equipment maintenance routine history, the equipment age, high running speed of the equipment, and the type of foundation. We discovered that some of this equipment in use has spent more than 50 years on operation, and their design does not really satisfy good ergonomic standards; it is believed that this also contributes to the high levels of WBV and HAV exposure of the operators in most of the tobacco companies.

## 5. Conclusion

The research was conducted to examine, evaluate, and analyze the ergonomic effect of extreme exposure to vibration on the operators to WBV and HAV in a tobacco industry. Exposure to whole-body vibration increases discomfort rate and sickness and often reduces the production volume, i.e., the efficiency of both the operators and the equipment, and high vibration intensity also increases equipment wear and tear rate; in order to avoid all this, evaluation of the vibration exposure on the production floor of SMD and PMD departments is essential. This study revealed that most operators in the tobacco industry are exposed to both WBV and HAV with values exceeding the EAV and ELV recommendations by the ISO standards and European Union Parliament Council [9], implying that all the operators are exposed to high intensity of daily exposure limits in all the shift patterns, hence prone to health hazards such as joints pain, sudden stroke attack while working on the machine, psychological imbalance, white patches or spot on their hands, and possibly an adverse effect on the digestion system and cardiovascular coordination systems, which can affect the musculoskeletal system of the body and could weaken the flexible cartilage (intervertebral) discs, which can cause back, neck illness, and even paralysis. This effect can be minimized by installing the equipment on the damped foundation, persistence wear rate check on the equipment, and prompt routine maintenance check on the equipment; the equipment should not be run at the maximum speed, a set speed at which the optimum volume can be met should be determined, operators should be allowed to do a thorough medical examination at least twice in a year to examine the extent of vibrational effects on them, and up-to-date training of the operator should be allowed to educate them on the consequence of vibration hazards including adequate information on the likely source of the vibration. Lastly, there should be a strict law to ensure total compliance by the tobacco company management with the daily dosage exposure limit to ascertain at what span an operator should be exposed to WBV and HAV while at work.

## Figures and Tables

**Table 1:** Vibration magnitudes (m/s^2^) corresponding to the hand-transmitted vibration exposure action and exposure limits in accordance to the Directive of the European Union [21].

Exposure duration	Exposure action value (m/s^2^)	Exposure limit value (m/s^2^)
1 s	424.26	848.53
10 s	134.16	268.33
1 minute	54.77	109.54
10 minutes	17.32	34.64
1 hour	7.07	14.14
2 hours	5.00	10.00
4 hours	3.54	7.07
8 hours	2.50	5.00
12 hours	2.04	4.08
16 hours	1.77	3.54
24 hours	1.44	2.89

**Table 2:** Types of production equipment used and their functions.

Equipment's	Numbers	Type	Speed	Maintenance type
GD121	2	Maker	15,000 rpm	Periodic maintenance, once a month
Tray filler	2	Maker	7500 rpm	Running maintenance
X3-X4	2	Packer	400 rpm	Running/breakdown maintenance
Filter loader	2	Maker	1500 rpm	Periodic/breakdown maintenance
Fork lift	2	CTS	4.06 m/s	Periodic maintenance
Tobacco cutter	2	Maker	5000 rpm	Periodic maintenance
T-10	2	Maker	3500 rpm	Periodic/running maintenance
Tobacco tipper	2	Maker	5 tip/min	Periodic maintenance
Leaf boiler	2	Maker	Optional	Periodic maintenance

**Table 3:** Illustration of the exposure action value, limit value, and health guidance for whole-body vibration in accordance to [14, 15, 20, 21] standard.

Exposure/HGCZ	ISO 2631-1(1997)		EU Directive 2002		ISO 2631-5	
	WRMS	VDV	WRMS	VDV	Sed	*R*
EAV/HGCZ lower limit	0.43 m/s^2^	8.5 m/s^1.75^	0.50 m/s^2^	9.1 m/s^1.75^	0.50 MPa	0.80
ELV/HGCZ upper limit	0.86 m/s^2^	17 m/s^1.75^	1.15 m/s^2^	21 m/s^1.75^	0.80 MPa	1.20

**Table 4:** Weighted RMS levels against both the B1 and B2 assessment limits in [21].

Limits	Exposure time (*T*_*e* _ <600)	*T*_*e*_ >600 s
B1 upper limit	6.0	6.00 *∗* (600/*T*_*e*_)^1/2^
B1 lower limit	(B1 upper limit)/2	(B1 upper limit)/2
	*T*_*e*_ <30 seconds	*T*_*e*_ >30 seconds
B2 upper limit	5.1885	5.1885 *∗* (30/*T*_*e*_)^1/4^
B2 lower limit	(B2 upper limit)/2	(B2 upper limit)/2

**Table 5:** Weighted RMS level and health risk assessment by [21].

Weighted RMS level	Health risk assessment
Below the limit	Unknown
Between the limit (caution zone)	Possible
Above the limit	Likely

## Data Availability

The data related to the research work can be provided upon request to the corresponding author.

## References

[1] Griffin M. J. (1990). *Handbook of Human Vibration London*.

[2] Griffin M. J. (2007). Negligent exposures to hand-transmitted vibration. *International Archives of Occupational and Environmental Health*.

[3] Troup J. D. (1979). Driver’s backpain and its prevention: a review of the postural, vibratory and muscular factors, together with the problem of transmitted road-shock. *Journal of Sound and Vibration*.

[4] Wolfgang R., Burgess-Limerick R. (2014). Whole-body vibration exposure of haul truck drivers at a surface coal mine. *Applied Ergonomics*.

[5] Bovenzi M. (2006). Health risks from occupational exposures to mechanical vibration. *La Medicina del Lavoro*.

[6] Bureau of Labor Statistics and USBOL, Nonfatal occupational injuries and illnesses requiring days away from work. Book nonfatal occupational injuries and illnesses requiring days away from work, 2015, https://www.bls.gov/news.release/osh2.toc.htm

[7] Zhao X., Schindler C. (2014). Evaluation of whole-body vibration exposure experienced by operators of a compact wheel loader according to ISO 2631-1 : 1997 and ISO 2631-5 : 2004. *International Journal of Industrial Ergonomics*.

[8] Mansfield N. J. (2004). *Human Response to Vibration New York*.

[9] E. U. Parliament, Directive 98/37/EC of the European Parliament Council on the approximation of the laws of the member states relating to machinery OJL 207, 1998

[10] Rom W. N., Markowitz S. B. (2007). *Environmental and Occupational Medicine*.

[11] Mansfield N., Sammonds G., Nguyen L. (2015). Driver discomfort in vehicle seats-effect of changing road conditions and seat foam composition. *Applied Ergonomics*.

[12] Punnett L. G., Park J.-S., Punnett L. (2006). Work routinization and implications for ergonomic exposure assessment. *Ergonomics*.

[13] Jahncke H., Hygge S., Mathiassen S. E., Hallman D., Mixter S., Lyskov E. (2017). Variation at work: alternations between physically and mentally demanding tasks in blue-collar occupations. *Ergonomics*.

[14] ISO : 2631-1 (1997). *Mechanical Vibration and Shock—Evaluation of Human Exposure to Whole-Body Vibration—Part 1: General requirements*.

[15] ISO : 2631-5 (2004). *Mechanical Vibration and Shock–Evaluation of Human Exposure to Whole-Body Vibration–Part 5: Method for Vibration Containing Multiple Shocks*.

[16] Milosavljevic S., Bergman F., Rehn B., Carman A. B. (2010). All-terrain vehicle use in agriculture: exposure to whole body vibration and mechanical shock. *Applied Ergonomics*.

[17] Lewis C. A., Johnson P. W. (2012). Whole-body vibration exposure in metropolitan bus drivers. *Occupational Medicine*.

[18] Edger T. (2006). Wholebody vibration exposure experienced by mining equipment operators. *Occupational Ergonomics*.

[19] Kittusamy N. K., Buchholz B. (2004). Whole-body vibration and postural stress among operators of construction equipment: a literature review. *Journal of Safety Research*.

[20] Eurofound, Six European Working Conditions Survey, 2015, https://www.eurofound.europa.eu/surveys/european-working-conditions-surveys/sixtheuropean-working-conditions-survey-2015

[21] Directive: 2002/44/EC, Vibration of 25 June 2002 on the Minimum Health and Safety Requirements Regarding the Exposure of Workers to the Risks Arising from Physical Agents (Vibration) (Sixteenth Individual Directive within the Meaning of Article 16(1) of Directive 89/391/EEC), 2002

[22] Sheth J., Mistri M., Pancholi D. (2019). Gaucher disease: single gene molecular characterization of one-hundred Indian patients reveals novel variants and the most prevalent mutation. *BMC Medical Genetics*.

[23] ISO : 5349-2 (2001). *Mechanical Vibration-Measurement and Evaluation of Human Exposure to Hand-Transmitted Vibration-Part 2: Practical Guidance for Measurement at the Workplace*.

[24] Butkiewicz M., Wang Y., Bryant S. H., Lowe E. W., Weaver C. D., Meiler J. (2017). High-throughput screening assay datasets from the PubChem database. *Chemical Informatics*.

